# The costs of extra‐pair behaviours in birds

**DOI:** 10.1002/brv.70178

**Published:** 2026-04-30

**Authors:** Jørgen S. Søraker, Jamie Dunning

**Affiliations:** ^1^ Edward Grey Institute of Field Ornithology, Department of Biology University of Oxford Oxford OX1 3EL UK; ^2^ Department of Biology Norwegian University of Science and Technology (NTNU) Trondheim N‐7491 Norway; ^3^ Imperial College London Silwood Park Campus is Buckhurst Road, Sunninghill, Ascot Berkshire SL5 7PY UK

**Keywords:** sexual selection, reproduction, extra‐pair paternity, fitness costs, parental care, sexually transmitted diseases, harassment, birds

## Abstract

Extra‐pair behaviours – reproductive behaviours, including those related to copulation and paternity of offspring, amongst animals outside of a social pair bond – have long intrigued behavioural ecologists, particularly from the female animal's perspective. Female extra‐pair paternity is not supported by classic models of sexual selection yet is common across birds. Researchers recognise that variation in extra‐pair paternity, both within and between species, is in part driven by the costs to the individual; however, empirical studies tend to focus on benefits. This is because benefits are often measured through direct fitness measurements, whereas costs have more complex, indirect pathways to fitness. However, both the prevalence and magnitude of a cost are experienced in the context of an individual's environment and may affect fitness, either directly (by affecting reproduction or survival) or indirectly (through the fitness of offspring). Here, we review our current understanding of costs associated with extra‐pair paternity and extra‐pair copulation. For example, we review both the costs of producing extra‐pair offspring and the behaviour associated with extra‐pair paternity. We conclude that the costs of extra‐pair paternity and extra‐pair copulation are understudied, but are likely a key factor shaping this behaviour. More research, particularly empirical and experimental studies in taxa other than birds, is needed to understand the intricate cost–benefit equation underlying extra‐pair paternity.

## I. INTRODUCTION

Extra‐pair behaviours, reproductive behaviours occurring outside of a social pair bond, ranging from copulation to offspring paternity, are common across the animal kingdom, with examples in mammals (Cohas & Allainé, 2009; Macdonald *et al*., 2019), reptiles (Bull, Cooper & Baghurst, 1998), fish (Coleman & Jones, 2011), and birds (Griffith, Owens & Thuman, 2002; Brouwer & Griffith, 2019). Even socially monogamous species (involving a single male and female pair) are often genetically polygamous, with one individual mating with multiple partners (Davies, Krebs & West, 2012). Passerine birds are a popular system for studying the drivers and fitness consequences of extra‐pair behaviours. They have annual reproductive cycles, often producing many offspring, and dynamic social systems (Griffith *et al*., 2002; Westneat & Stewart, 2003; Kempenaers & Schlicht, 2010; Brouwer & Griffith, 2019). Extra‐pair paternity has been recorded in around 75% of species otherwise regarded as socially monogamous (Brouwer & Griffith, 2019).

Theoretically, extra‐pair behaviours are explained through evolutionary advantage to parents or, indirectly, to offspring (Trivers, 1974; Arnqvist & Kirkpatrick, 2005). For example, through extra‐pair paternity, males can increase the number of offspring produced with a breeding period without investing in costly parental care, providing an obvious fitness advantage (Dunning *et al*., 2024). However, the motivation for females to engage in extra‐pair copulations, and to raise extra‐pair offspring, is less obvious. It was an early suggestion that, because females are limited to a finite number of eggs over their lifetime, they should invest extra‐pair copulations only with males of higher quality than their social partner, therein providing indirect benefits to offspring and her own reproductive success (Trivers, 1974; Queller, 1997). However, empirical demonstrations of such indirect benefits to females are scarce (Westneat, Sherman & Morton, 1990; Arnqvist & Kirkpatrick, 2005; Akçay & Roughgarden, 2007; Forstmeier *et al*., 2014; Reid *et al*., 2014; Arct, Drobniak & Cichoń, 2015).

Several, often conflicting, hypotheses have been proposed to explain female engagement in extra‐pair behaviour. Because extra‐pair behaviours are often considered from the perspective of providing an evolutionary advantage, these hypotheses often focus on the benefits to the female (Birkhead & Møller, 1992; Griffith *et al*., 2002; Brouwer & Griffith, 2019). These hypotheses are therefore broadly divided into direct and indirect benefits, of which both could result in extra‐pair behaviour as an adaptive reproductive strategy for females. Direct benefits of extra‐pair behaviour for females would increase their reproductive success or survival. For example, extra‐pair males may provide insurance against social mate infertility (Sheldon, 1994; Wetton & Parkin, 1997; Santema, Teltscher & Kempenaers, 2020; Vedder, 2022). Other direct benefits may include access to resources (Wolf, 1975; Gray, 1997) and increased parental care or nest defence (Townsend, Clark & McGowan, 2010; Mennerat *et al*., 2018; Krams *et al*., 2022). Although such interactions would directly benefit female fitness, support for such benefits are limited (Arnqvist & Kirkpatrick, 2005; Mennerat *et al*., 2018) and so, are unlikely to explain the considerable variation in rates of extra‐pair paternity alone. Conversely, indirect benefit (or ‘good genes’) hypotheses suggest that female reproductive fitness is increased *via* improved offspring fitness. For example, a female may increase the genetic quality of her offspring by reproducing with a male of better, (Westneat *et al*., 1990; Birkhead & Møller, 1992; Tregenza & Wedell, 2000; Griffith & Immler, 2009) or more compatible genetic quality (i.e. through inbreeding avoidance; Hansson, Hasselquist & Bensch, 2004) than her social partner. However, despite considerable empirical research and meta‐analyses on the subject (Arnqvist & Nilsson, 2000; Akçay & Roughgarden, 2007), extra‐pair behaviours seldom provide indirect benefits to females in wild systems (Akçay & Roughgarden, 2007; Sardell *et al*., 2012), and, often suggest costs, either to females (Matysioková & Remeš, 2013; Schroeder *et al*., 2016) or to extra‐pair offspring (Schmoll *et al*., 2009; Sardell *et al*., 2012; Hsu *et al*., 2014). Despite this apparent lack of an obvious benefit to females or their offspring from extra‐pair behaviours, females actively seek extra‐pair copulations (Griffith *et al*., 2002; Westneat & Stewart, 2003; Griffith & Immler, 2009; Brouwer & Griffith, 2019), presenting a paradox: for what reason do female animals seek extra‐pair copulation?

More recent, sexual conflict hypotheses have considered how, under certain conditions, extra‐pair behaviour may persist whilst maladaptive for females. This could happen where (*i*) extra‐pair paternity is adaptive for males, and male benefits outweigh the costs to females – referred to as the intersexual pleiotropy hypothesis (Wang *et al*., 2020; Dobson *et al*., 2023) – or (*ii*) where genes for other, unrelated traits under positive selection also contribute to the expression of extra‐pair behaviour – the intrasexual pleiotropy hypothesis (Halliday & Arnold, 1987). Unlike benefit focused hypotheses, sexual conflict hypotheses focus on the disadvantages to females relative to the benefits experienced by males (Hsu *et al*., 2014). Sexual conflict hypotheses have been difficult to test empirically, due to incomplete description of genetically linked behaviours, but also, incomplete assessment of the magnitude of costs (Lifjeld *et al*., 1993; Maher *et al*., 2017) and have mostly been supported by indirect evidence (Jouventin *et al*., 2007; Sardell *et al*., 2011). Thus, although sexual conflict hypotheses demonstrate that extra‐pair behaviours could be maintained despite being maladaptive to females, measuring the relative costs is key to empirical evaluation.

Although several costs of extra‐pair behaviour have been described both for males and females (listed in Tables 1 and 2), they are scarcely studied experimentally (Komdeur, 2001; Low, 2006; Dunn & Whittingham, 2007) and difficult to demonstrate empirically without large sample sizes and precise fitness data (Forstmeier *et al*., 2014). Empirical studies therefore focus on identifying the reproductive benefits of extra‐pair paternity, but they rarely access (or are aware of) the costs in the same context. Although quantifying benefits is easier than quantifying costs, studies often also fail to consider them. For example, although obtaining extra‐pair paternity can increase the number of offspring for males (Raj Pant *et al*., 2022; Dunning *et al*., 2024), seeking extra‐pair copulation also comes with search costs (Dunn & Whittingham, 2007) and leaves the female unguarded in the same period, hence risking loss of within‐pair paternity (Møller & Ninni, 1998; Harts & Kokko, 2013). Therefore, under‐representation or underestimation of the costs may bias our understanding of extra‐pair behaviours.

**Table 1:** The costs associated with extra‐pair paternity, gaining extra‐pair copulations and avoiding extra‐pair copulations for females.

Type	Cost	Direct or indirect	Reference
Cost of extra‐pair paternity	Reduced paternal care	Both	Møller & Cuervo (2000); Suter *et al*. (2009); Schroeder *et al*. (2016); Søraker *et al*. (2023)
	Reduced genetic quality	Indirect	Townsend *et al*. (2009); Sardell *et al*. (2011)
	Increased sibling competition	Both	Briskie *et al*., (1994)
	Divorce	Direct	Ramsay *et al*. (2000); Dunning *et al*. (2023)
Cost of gaining extra‐pair copulations	Sexually transmitted diseases (STDs)	Direct	Sheldon (1993); Westneat & Birch Rambo (2000)
	Cost of acquiring partner	Direct	Dunn & Whittingham (2007); Kenny‐Duddela *et al*. (2025)
	Harassment from social male	Direct	Valera *et al*. (2003)
Cost of avoiding extra‐pair copulations	Harassment from extra‐pair male	Direct	McKinney & Evarts, (1998); Wysocki *et al*. (2025)

**Table 2:** Summary of the costs to the males associated with extra‐pair paternity, gaining extra‐pair copulations and avoiding the female from having extra‐pair copulations.

Type	Cost	Direct or indirect	Reference
Cost of extra‐pair paternity	Reduced paternity	Direct	Westneat *et al*. (1990); Griffith *et al*. (2002)
Cost of gaining extra‐pair copulations	Sexually transmitted diseases (STDs)	Direct	Sheldon (1993); Westneat & Birch Rambo (2000)
	Cost of acquiring partner	Direct	Dunn & Whittingham (2007); Kenny‐Duddela *et al*. (2025)
Cost of avoiding female extra‐pair copulations	Mate guarding	Direct	Komdeur (2001); Kokko & Morrell (2005); Dowling & Webster (2017)

Costs of extra‐pair behaviour can take different pathways. Like benefits, discussed above, they can be direct (Komdeur, 2001) or indirect (Hsu *et al*., 2014). A direct cost reduces fitness through reduced reproduction or survival, whereas indirect costs affect the fitness of offspring, for example through lowered genetic quality (Townsend *et al*., 2010; Sardell *et al*., 2011; Hsu *et al*., 2014). Direct and indirect costs are not mutually exclusive, as certain mechanisms can affect both parental and offspring fitness at the same time. Further, costs of extra‐pair behaviour, both direct and indirect, will also depend upon the mating system in the population. For example, males might benefit from accepting lost within‐pair paternity if this constitutes doing the best of a bad job (Greene *et al*., 2000). Hence, the costs should always be evaluated in the mating context of the studied population.

Here, we review the costs of extra‐pair behaviour, splitting them into costs associated with extra‐pair copulation and raising extra‐pair paternity. Although costs are mostly associated with female fitness, we have also included costs to males where they are identified. In addition, while we primarily focus on birds, many of the reviewed costs are also relevant for other taxa like mammals, reptiles, insects and fish. This is also true for mating systems other than social monogamy. We suggest that re‐framing the question of why extra‐pair behaviours persist in the context of costs, as well as fitness advantages, will improve our understanding of the drivers of extra‐pair behaviours, particularly from the female perspective.

## II. THE COST OF EXTRA‐PAIR PATERNITY

There are multiple potential costs of producing extra‐pair offspring, both from the male and female perspectives. Here, we review the costs of extra‐pair paternity. For females, reduced paternal care, reduced offspring fitness, increased sibling competition and social divorce are reviewed costs (Table 1). For males, the core cost is reduced paternity (Table 2). Here, we first review the costs for females (F), before we move on to the costs for males (M).

### (1) Reduced parental care (F)

Reduced parental care caused by lowered paternity is an intensively studied cost of extra‐pair paternity (Møller & Birkhead, 1993; Schwagmeyer *et al*., 1999; Møller & Cuervo, 2000; Schroeder *et al*., 2016; Søraker *et al*., 2023), because parental care is known to be particularly costly (Santos & Nakagawa, 2012). Reduced paternal care, imposed by the male is directly costly for the female (Weatherhead *et al*., 1994). Theoretical models show mixed predictions for adaptive responses in care related to paternity (Sheldon, 2002). This is partly due to differing assumptions in the models. For example, a key component in some models is the male's ability to assess paternity (Sheldon, 2002), which is still debated in birds (Pagel, 1997; Johnstone, 1997). Theoretical models also highlight the importance of the level at which extra‐pair paternity is measured (i.e. individual level *versus* population‐level). Individual costs of extra‐pair paternity evaluate adaptive responses to varying levels of paternity that happen within an individual's lifespan (i.e. at ecological timeframes) (Schroeder *et al*., 2016; Brouwer & Griffith, 2019). A cost of adaptive within‐male adjustment to extra‐pair paternity will be a direct cost as it calls for compensation by the female. However, it can also be an indirect cost if the female does not fully compensate, potentially resulting in lowered offspring quality (Markman, Yom‐Tov & Wright, 1995). Such within‐male variation in within‐pair paternity levels can be driven by male quality. In such cases, low‐quality males with low level of paternity may be left with no better option than maintaining the same level of paternal care. Therefore, we might not expect the level of parental care to decrease with extra‐pair paternity among individuals of the same population (Westneat & Stewart, 2003). Selection over generations can, however, result in species‐level responses in an evolutionary timeframe (Wright, 1998). Among‐species studies of extra‐pair paternity in this context mostly consider the change in average level of care in relation to the average species‐level extra‐pair paternity‐rate (Møller & Birkhead, 1993; Schwagmeyer *et al*., 1999; Møller & Cuervo, 2000; Søraker *et al*., 2023). However, such evolutionary relationships do not provide evidence of within‐individual adjustments to extra‐pair paternity in their social clutch.

Evidence from empirical studies of both among‐species and individual responses to reduced paternal care are mixed (Møller & Birkhead, 1993; Schwagmeyer *et al*., 1999; Møller & Cuervo, 2000; Bouwman, Lessells & Komdeur, 2005). Among‐species investigations generally show that males decrease the level of care with reduced paternity, although it is still debated which forms of care are reduced (Møller & Birkhead, 1993; Schwagmeyer *et al*., 1999; Møller & Cuervo, 2000; Søraker *et al*., 2023). Different forms of care have different implementations (Arnqvist & Kirkpatrick, 2005). While some studies find pre‐hatching forms of care to be affected by paternity (e.g. incubation, nestbuilding etc.; Matysioková & Remeš, 2013; Lifjeld *et al*., 2019; Søraker *et al*., 2023), other studies support post‐hatching care (e.g. provisioning) being affected by paternity (Møller & Birkhead, 1993; Schwagmeyer *et al*., 1999; Søraker *et al*., 2023).

Studies on individual costs of extra‐pair paternity show weaker consistency than among‐species costs. Several studies have been performed linking paternity and paternal care, showing support for both a reduction in care (Lifjeld, Slagsvold & Ellegren, 1998; Sheldon & Ellegren, 1998; Suter *et al*., 2009; Schroeder *et al*., 2016), and no effect on care (Westneat, 1995; Yezerinac, Weatherhead & Boag, 1996; Bouwman *et al*., 2005; Barati *et al*., 2018; Poblete *et al*., 2021). In reed buntings *Emberiza schoeniclus*, Suter *et al*. (2009) showed that the males reduced provisioning rate in response to decreased paternity. This reduction was further compensated by the female, indicating a direct cost of extra‐pair paternity for the female. However, Bouwman *et al*. (2005) did not find any evidence for such a negative relationship between levels of extra‐pair paternity and paternal care in the same species. An among‐species investigation of within‐male adjustment across several taxa found no effect of paternity on parental care (Griffin, Alonzo & Cornwallis, 2013). However, reduction in male care were more likely if the cost of caring and risk of reduced paternity were high (Griffin, Alonzo & Cornwallis, 2013). Several of these investigations have been experimental, and cross‐fostering experiments are common. Cross‐fostering experiments are, however, seldom informative as the cues that males might use to assess paternity are poorly understood (Kempenaers & Sheldon, 1997).

Due to mixed evidence on the individual costs of extra‐pair paternity on parental care, it is hard to extract general patterns (Bouwman *et al*., 2005). However, most studies fail to address concerns on important confounding factors, like environmental variation, partner quality and an individual's physical state (Kempenaers & Sheldon, 1997). For example, even when a decrease in care with paternity is described, it is often difficult to disentangle the cause of reduced care. If low quality males experience lower levels of paternity, it is also challenging to know if the decreased care is due to their state or the level of care (Griffith *et al*., 2002). Another important factor to consider, both in among‐individual and among‐species studies, is the trade‐off between male care and seeking extra‐pair paternity for himself. This can affect the optimal level of care both for low‐quality and high‐quality males, although it is likely more apparent for high‐quality males (Werren, Gross & Shine, 1980; Houston & McNamara, 2002). Costs to the female of reduced paternal care may also be dependent on female state, and studies linking state‐dependency to cost of extra‐pair paternity and female fitness components would provide helpful insight (Gowaty & Bridges, 1991).

Schroeder *et al*. (2016) studied paternal incubation and provisioning in house sparrows *Passer domesticus*, while controlling for several of these confounding effects (Kempenaers & Sheldon, 1997). They disentangled within‐ *versus* among‐male differences in care related to varying paternity. The study found that females consistently differed in their individual extra‐pair paternity‐rate. Males reduced provisioning only when mated with a female consistently engaging in extra‐pair paternity. Therefore, males adjusted their level of care to female identity, rather than the paternity‐level in the clutch. A cross‐fostering experiment suggested that males did not adjust provisioning to paternity, although this suggests that they are unable to assess nestling paternity. To fully understand the role of paternity on parental care, more studies accounting for confounding factors (Kempenaers & Sheldon, 1997) are needed. Further, disentangling within and among‐male adjustments are informative to understand the potential (and confounding) effect of difference in male ‘quality’ and how it relates to a male's response to loss in paternity.

The study of reduced parental care as a cost of extra‐pair paternity is therefore complex. However, few have applied statistical methods that clearly account for confounding factors. Future studies should include factors like pair‐constitution, environmental variation, individual state and whether the measured trait (for example provisioning rate) is a reliably proxy for parental care. Moreover, combining within‐ and among‐species data could, in a comparative framework, inform the relative importance of ecological *versus* evolutionary processes as drivers of variation in parental care.

### (2) Reduced genetic quality (F)

Increased genetic quality inherited from extra‐pair males is one of the main hypotheses driving female extra‐pair paternity (Westneat *et al*., 1990; Akçay & Roughgarden, 2007). However, when mating with extra‐pair partners, females also risk reducing the genetic quality of their offspring (Forstmeier *et al*., 2014). Females probably indirectly assess the genetic quality of extra‐pair mates, for example *via* secondary sexual traits (Sheldon & Ellegren, 1998; Griffith *et al*., 2002). Although these traits are often found to be reliable indicators of male quality (Andersson & Simmons, 2006), it could also reduce the female's ability to sample the quality of potential mates sufficiently if these are selected for other reasons than male quality (Lifjeld & Slagsvold, 1997). Uncertainty in accessed male quality can therefore result in sub‐optimal assessment of fitness of both the social and the extra‐pair partner (Hasson & Stone, 2011). Thus, females could reduce the genetic quality of the offspring through extra‐pair paternity. Sperm choice, where females choose between sperm from several mated males is thought to partly solve this problem (e.g. choosing only social males sperm), but evidence for differential sperm selection by females in limited (Birkhead & Møller, 1992).

Analysis of the genetic quality of offspring needs to be rooted in one or more fitness components. Multivariate analysis now allows for interpretation of covariance between multiple fitness components (Dingemanse & Dochtermann, 2013), but different components measure subtly different aspects of individual fitness. Some studies suggest that lifetime reproductive success may be the optimal fitness estimate (Sardell *et al*., 2011; Hsu *et al*., 2014), but cost and benefits are often only apparent in the short term (Alif *et al*., 2022). However, cases may arise where it is natural to look to other fitness components, particularly if one has detailed knowledge of fitness measurements in the population (Alif *et al*., 2022), and *a priori* hypotheses are made about certain fitness relationships.

While most studies find no difference between extra‐pair offspring and within‐pair offspring in measurable fitness components (Akçay & Roughgarden, 2007), some studies find extra‐pair offspring to have higher fitness than within‐pair offspring, for example in blue tits *Cyanistes caeruleus* (Foerster *et al*., 2003) and dark‐eyed junco *Junco hyemalis* (Gerlach *et al*., 2012). However, a few studies find extra‐pair offspring have reduced fitness than within‐pair offspring (Sardell *et al*., 2011). In house sparrows, extra‐pair offspring had lower recruitment probability and produced fewer fledgelings than within‐pair offspring (Hsu *et al*., 2014). Further, in song sparrows *Melospiza melodia*, recruitment and lifespan did not differ between extra‐pair offspring and within‐pair offspring (Sardell *et al*., 2011). However, this study demonstrated a sex‐specific genetic cost of extra‐pair offspring, with female, but not male, extra‐pair offspring having lower recruitment and shorter lifespan than within‐pair females. The opposite sex‐effects were found for the coal tit *Periparus ater*, where male, but not female, within‐pair offspring had higher numbers of social offspring throughout their life (Schmoll *et al*., 2009). These studies underpin the important aspect of sex‐dependency when comparing extra‐pair offspring and within‐pair offspring. Environmental conditions could also affect the costs of producing extra‐pair offspring, just as it can affect the benefits (Yang *et al*., 2024). Although contrary findings to these results exist (Foerster *et al*., 2003; Akçay & Roughgarden, 2007; Gerlach *et al*., 2012), they do show that producing extra‐pair offspring can lower fitness under certain circumstances.

While studying American crows *Corvus brachyrhynchos*, Townsend *et al*. (2010) found that extra‐pair offspring were more inbred than within‐pair offspring, and that inbreeding lowered survival in this population (Townsend *et al*., 2009), illustrating an indirect cost of extra‐pair paternity. There can also be population‐level costs, such as lowered effective population size, reduced evolvability, reduced genetic variation (see Section II.5) and population‐level inbreeding depression, with potential severe consequences also at population level.

Reduced genetic quality of offspring is likely a restricted cost of extra‐pair paternity. Indeed, of the mentioned examples, only one showed an effect on the reproductive fitness of offspring (Hsu *et al*., 2014). However, this is expected if reduced offspring genetic quality is a large cost of extra‐pair paternity, and not all poor‐quality offspring die prior to recruiting into the breeding population.

### (3) Increased sibling competition (F)

Increased sibling competition is rarely considered as a cost of extra‐pair paternity (Forstmeier *et al*., 2014). However, it follows from theories of parent–offspring conflict, sibling competition and the effects of reduced within‐family relatedness (Trivers, 1974; Royle, Hartley & Parker, 2002). While such increased competition may reduce the indirect fitness of the male (increased competition for his offspring), it is primarily a cost to the female, where all her offspring are affected.

Lower average relatedness, among the offspring and between the offspring and the parent, for example because of extra‐pair paternity, causes the optimal level of investment for each specific offspring to increase and the demand for parental investment to increase (Trivers, 1974). For example, begging intensity has been suggested to increase with increasing extra‐pair paternity‐rates. Intensified begging could result in increased parental care (Wright & Leonard, 2007) and could therefore represent a direct cost for the parent(s) that provide more than their optimum level of care. It could potentially also increase predation risk. Costs of increased sibling competition through begging could consider changes in begging intensity with variation in within‐clutch relatedness, but, although this has been intensively studied, it has rarely been demonstrated within species [as reviewed by Wright & Leonard (2007), but see Boncoraglio & Saino (2008) for an exception], while an effect is found among species (Briskie, Naugler & Leech, 1994). One reason why siblings do not adjust begging to relatedness could be the lack of stable paternity markers. Extra‐pair mates would be selected to avoid paternity markers, which could reveal their identity for the social mate. However, how paternity markers evolve, either genetically or through behaviour, is debated and topic for considerable theoretical modelling (Pagel, 1997; Johnstone, 1997).

### (4) Divorce rate (F)

Engagement in extra‐pair paternity may also increase the divorce rate (i.e. the rate at which social partners are changed across reproductive events). Such a relation among species has been demonstrated (Cezilly & Nager, 1995; Chen *et al*., 2023) but is rarely informative for individual costs. Divorce could be a direct cost for the individual, because more experienced pairs often have higher reproductive success (Ens, Safriel & Harris, 1993), and could result from a variety of reasons, for example low reproductive success in previous broods (Culina, Radersma & Sheldon, 2015; Dumas *et al*., 2025). Individuals engaging in extra‐pair copulations may experience a higher probability of divorce (Chen *et al*., 2023). Both males and females could initiate divorce but should only do so if they have better options. Hence, females divorcing the male based on his engagement in extra‐pair paternity is unlikely, especially if this does not affect his level of paternal care to the social brood. Therefore, divorce resulting from extra‐pair paternity would most likely be male‐initiated based on reduced paternity. Relationship between extra‐pair paternity and divorce has been demonstrated in black‐capped chickadees *Poecile atricapillus* (Ramsay *et al*., 2000), where females involved in extra‐pair copulations were more likely to switch partners between breeding events. However, there were no differences in reproductive success whether the female was involved in extra‐pair copulations or not. Females also divorced for males of higher rank, indicating that they divorced for a better option rather than as a result of being divorced by the male due to extra‐pair paternity. In house sparrows, females that switched partner more often were more likely to have extra‐pair offspring, although they did not have a higher proportion of extra‐pair offspring (Dunning, Burke & Schroeder, 2023) implying that the female did not obtain a benefit from the extra‐pair copulations (Wang *et al*., 2020; Dunning *et al*., 2023). Instead, extra‐pair copulation may be a linked trait with social copulation rate, possibly driving divorce indirectly (Dunning *et al*., 2023). The costs of divorce may also depend upon what sex would hold the territory after the divorce, which was male‐biased in alpine swifts *Tachymarptis melba* (Dumas *et al*., 2025) but is largely unknown for most species. Still, the most limiting factor for a fuller understanding of divorce as a cost of extra‐pair paternity is the scarceness of empirical investigations. However, many systems used to study extra‐pair paternity also contain data on social pair bonds, offering opportunities for further investigation.

### (5) Reduced paternity (M)

From the male perspective, loss of within‐pair paternity, where the female of the pair gains extra‐pair paternity outside of the pair bond, is the most apparent, and likely highest cost of extra‐pair paternity (Brouwer & Griffith, 2019). Loss of paternity is a direct cost of extra‐pair paternity as it reduces the number of offspring sired (Westneat & Stewart, 2003). Although studies have investigated the fitness benefits of obtaining extra‐pair paternity (Raj Pant *et al*., 2022), fewer have investigated reduced fitness due to loss of paternity – that is, reduced lifetime reproductive success. Several components need to be considered under such a scenario. First, loss of within‐pair paternity could be counteracted by extra‐pair paternity (Griffith *et al*., 2002), which could increase a male's reproductive success. Second, more complex mating systems can affect the relative cost of paternity loss. For example, brightly coloured lazuli bunting *Passerina amoena* favour territories with vegetation nest cover, and older males are aggressive towards younger males settling in neighbouring territories. Yet, older males gain more extra‐pair paternity from neighbours when dull yearlings settle (Greene *et al*., 2000). Hence, young males have a net benefit from settling with lower within‐pair paternity, which easily could be measured as costly. Third, from studying characteristics of males obtaining extra‐pair paternity and losing within‐pair paternity, we know that reduced paternity is dependent on both quality and age of the male. For example, males often obtain more extra‐pair paternity with age (Hsu *et al*., 2015), although the evidence for an effect of age on within‐pair paternity is mixed (Cleasby & Nakagawa, 2012; Raj Pant *et al*., 2022).

Although this review focuses on the costs of extra‐pair paternity to the individual, reduced paternity could also have population‐level consequences as it can affect both adaptation and population dynamics. For example, extra‐pair paternity can influence variance in reproductive success (Raj Pant *et al*., 2022), thereby affecting the effective population size (Lotterhos, 2011). In song sparrows, O'Connor *et al*. (2006) showed that extra‐pair paternity had a low effect on effective population size by comparing estimates from social and genetic data. Another way to investigate how extra‐pair paternity influences effective population size is to decompose demographic variance to understand its sensitivity to variation in reproductive success, but such empirical studies in birds are lacking (Lee *et al*., 2020). Increased variance in reproductive success caused by sexual selection may also affect the evolvability of a species due to reduced genetic variation, commonly referred to as the ‘lek paradox’ (Petrie & Roberts, 2007; Kotiaho *et al*., 2008; Dugand, Tomkins & Kennington, 2019). However, the potential role of extra‐pair paternity in the lek paradox through affecting sexual selection remains poorly understood. Nevertheless, in most species with extra‐pair paternity, extra‐pair males are often neighbours (Mennerat *et al*., 2018), so the skew in reproductive success is likely limited.

## III. THE COSTS OF GAINING EXTRA‐PAIR COPULATIONS

As both males and females potentially benefit from extra‐pair behaviour (Westneat & Stewart, 2003), both sexes might actively seek extra‐pair copulation. However, seeking extra‐pair copulation may come with costs that operate outside of realised paternity. For example, transmission of sexually transmitted diseases and the costs of acquiring an extra‐pair partner affect both sexes. In addition, females might experience sexual harassment from the social male if they engage in extra‐pair copulation.

### (1) Harassment from social male (F)

When engaging in extra‐pair copulation, females may experience physical harassment from their social partner. This can be a severe direct cost and could affect the timing and occurrence for extra‐pair copulation (Valera, Hoi & Krištín, 2003). Social males can harass their females, as deterrent ‘punishment’ for engaging in extra‐pair copulations. This was documented in lesser grey shrikes *Lanius minor*, where females performing extra‐pair copulations experienced aggression from social mates. Moreover, the study found that this harassment was not so costly for males (Valera *et al*., 2003). Still, females could reduce the cost of harassment, as extra‐pair copulations could be performed cryptically compared to within‐pair copulations, for example, in great grey shrike *Lanius excubitor*, within‐pair copulations were performed openly, while extra‐pair copulations took place in more vegetative areas (Antczak, Hromada & Tryjanowski, 2007). However, we lack demonstration of this extra‐pair copulation‐induced harassment in most species (Valera *et al*., 2003), both from observational and experimental studies, and harassment from the social male remains a poorly understood cost of extra‐pair behaviour.

### (2) Sexually transmitted diseases (F & M)

Pathogens can affect individual fitness (for both males and females), population dynamics (Hudson & Dobson, 1991; Anderson & May, 1992) and sexual selection (Hamilton & Zuk, 1982). As several pathogens can be transmitted during copulation, promiscuous behaviour has been suggested to increase the prevalence of sexually transmitted diseases (STDs; Thrall, Antonovics & Bever, 1997). This is also true outside of birds, and also for alternative mating systems. Sheldon (1993) defined an STD as ‘…any pathogen that is transmitted during the act of copulation’, including *via* the reproductive organs (Lombardo & Thorpe, 2000) or ectoparasites transmitted through physical contact, such as feather parasites (Hillgarth, 1996; Kulkarni & Heeb, 2007; Ruiz‐Rodríguez *et al*., 2009).

While STDs have been identified in multiple wild populations of birds (Lombardo & Thorpe, 2000; Poiani & Wilks, 2000; Westneat & Birch Rambo, 2000; White *et al*., 2010), we know remarkably little about the relationship between STDs and extra‐pair behaviour. Theory suggests that STDs could affect individual extra‐pair behaviour (Kokko *et al*., 2002) *via* their influence on individual fitness (Sheldon, 1993). An increasing number of sexual partners per individual is predicted to result in higher prevalence of STDs per individual (Poiani & Wilks, 2000), although social barriers like pre‐copulatory behaviour may reduce the probability transmission (Loehle, 1995). However, clear empirical consensus is lacking. Westneat & Birch Rambo (2000) studied the costs of extra‐pair paternity in red‐winged blackbirds *Agelaius phoeniceus* based on exposure to STDs and found no effect of STDs on male fertility or female reproduction in a relatively limited sample. Furthermore, they demonstrated high variation in identified pathogens among males, underpinning the importance of pathogen‐specific hypothesis testing. Another study found heterogeneity in pathogenic microbes among tree swallows *Tachycineta bicolor*, but this did not affect semen characteristics in the studied population (Lombardo & Thorpe, 2000). These studies suggest that costs associated with STDs may be low. However, more (preferably experimental) studies are needed to test predictions about how STDs relate to extra‐pair matings, for example the prediction that the rate of extra‐pair copulations will be lower in populations with higher rates of (harmful) STDs.

In recent years, researchers have also considered the role of symbiotic microbial communities living in or on wild birds. Microbiomes include pathogenic microbes with the potential to impact fitness, and methods for detection of such pathogens have improved in recent years (Caporaso *et al*., 2010). In the rufous‐collared sparrow *Zonotrichia capensis*, the cloacal microbiome changed during the breeding season for males, and were more diverse early in the breeding season, potentially because of sexual transmission during mating (Escallón, Belden & Moore, 2019). However, few have tested how microbiomes relate to extra‐pair paternity directly, and this certainly warrants further investigation (Caporaso *et al*., 2010; Escallón *et al*., 2019). Although caution must be used regarding the source sample (e.g. faecal sample, semen sample, etc.), this is a promising technique that could benefit future empirical research into STDs as costs of extra‐pair copulation.

Among species, a positive covariation between average extra‐pair paternity rate and the presence and prevalence of STDs is expected. In a comparison of four bird species with contrasting mating systems, STDs were more prevalent in more promiscuous species (Poiani & Wilks, 2000). However, this should not be regarded as evidence for a cost of extra‐pair behaviour, as this could reflect a co‐evolutionary effect between parasites and hosts (Lombardo, 1998). However, among‐species investigations are insightful to the potential defence mechanisms to reduce costs of STDs. For example, the uropygial gland in birds produces anti‐parasitic substances (Jacob & Ziswiler, 1982; Ruiz‐Rodríguez *et al*., 2009). A comparative analysis showed larger uropygial glands in species with higher levels of extra‐pair paternity, perhaps as an antiparasitic defence mechanism against the costs of extra‐pair paternity (Møller, Søraker & Soler, 2020). Bird species with higher levels of extra‐pair paternity also seem to have larger spleens, which are an important part of the immune system (Møller, 1997). These studies do not demonstrate a causal link between uropygial gland size, spleen size and extra‐pair paternity. Such covariation is more likely to be due to more general immune responses that are not causally driven by extra‐pair paternity. Empirical studies within precocial species to explore these relationships would be interesting.

### (3) Costs of acquiring extra‐pair mates (F & M)

The costs of acquiring an extra‐pair mate have traditionally been split into search cost (i.e. the cost of physically searching for an extra‐pair partner; Dunn & Whittingham, 2007), and the energetically costly display for extra‐pair partners (Lifjeld & Slagsvold, 1997). However, these costs have received little empirical attention, particularly experimentally (Dunn & Whittingham, 2007). These costs have been proposed under a system of adaptive extra‐pair paternity (Birkhead & Møller, 1992; Dunn & Whittingham, 2007), and are experienced by either sex, affecting the same individual (i.e. the individual seeking extra‐pair copulation).

Search costs can be viewed as a time cost, lost opportunity cost and/or an energetic cost. The travel distances required to find potential extra‐pair partners can be extensive (Buitron, 1983; Currie *et al*., 1998) but the energetic expenditure is expected to be low (Dunn & Whittingham, 2007). Indeed, extra‐pair partners are often neighbours (Mingju *et al*., 2017; Mennerat *et al*., 2018; Beck, Valcu & Kempenaers, 2020; Beck, Farine & Kempenaers, 2020a), further reducing the net cost of this behaviour. Experimental evidence suggests that search cost may affect the distribution, but not the level, of extra‐pair paternity. Dunn & Whittingham (2007) experimentally increased search costs by cutting wing‐feathers of female tree swallows *Tachycineta bicolor* and found similar extra‐pair paternity rates for wing‐cut *versus* control females. However, wing‐cut females had more local extra‐pair partners, but the researchers were unable to disentangle the causal patterns of the observed variation in extra‐pair paternity (Dunn & Whittingham, 2007). Such a result could be due to females seeking extra‐pair copulation closer to their local area, but it could also be because they were less able to resist copulations by coercion from other males. This is also supported in barn swallows *Hirundo rustica*, where female space use was correlated with extra‐pair paternity in replacement clutches (Kenny‐Duddela *et al*., 2025). Search costs may also be the reason why several studies find population density to be a determinant of extra‐pair paternity rates (Westneat & Stewart, 2003) because higher densities could reduce search costs.

While searching for an extra‐pair partner, time spent away from the nest also increases the risk of others stealing nest material (Helfenstein *et al*., 2004). Although this is an untested hypothesis, stealing of nest material has been reported in multiple bird species (Siegfried, 1971; Cooper, 1986), and may be a relevant cost of extra‐pair behaviour that merits further investigation. Further, this time away from the nest also makes it prone to brood parasitism (Payne & Sorensen, 2005). However, no study has linked the risk of brood parasitism, both inter‐, and intra‐specific, to extra‐pair behaviour through this mechanism specifically. When an extra‐pair partner is located, courtship displays towards this potential partner may be required (Lifjeld & Slagsvold, 1997). While courtship displays have time and energetic costs, these are relatively small (Clark, 2012).

## IV. THE COSTS OF AVOIDING EXTRA‐PAIR COPULATIONS

There are also scenarios when either sex would avoid extra‐pair copulations. To ensure within‐pair paternity, males (M) might engage in mate guarding. However, the female (F) might also be interested in mating only with the social male because she wants to avoid the costs her social male may enforce. Thus, when the female wants to avoid extra‐pair copulation, they may experience harassment from potential extra‐pair males.

### (1) Harassment from extra‐pair partner (F)

Females may experience harassment from potential extra‐pair mates. This harassment can result in female injury, or even death (McKinney & Evarts, 1998). Extreme examples of harassment are comparatively rare in birds, and they are mostly recorded within waterfowl and the stitchbird *Notiomystis cincta* (Low, 2008; Adler, 2010), although it is also reported for a variety of other birds (McKinney & Evarts, 1998; Hooper *et al*., 2024). Although not strictly tested, harassment from extra‐pair males has been reported to result in death, injury, reduced fertility and abandoning breeding attempts [single reports reviewed by McKinney & Evarts (1998)]. In the stitchbird, harassment from extra‐pair males is suggested to affect egg‐laying patterns, as harassment could drastically decrease female state (Low, 2008). The risk of harassment could also lead to energetic costs, as avoiding harassment often include movement (McKinney & Evarts, 1998). Indeed, female blackbirds *Turdus merdula* have been shown to reduce forced extra‐pair copulations though changing their home ranges (Wysocki, Cholewa & Halupka, 2025), which potentially could affect other behaviours like foraging etc. This suggests that harassment can have an evolutionary impact on extra‐pair behaviours. It has been suggested that harassment and forced copulations from extra‐pair males also can infer stress on the female and affect her incubation (Gill *et al*., 2020). Extra‐pair copulations can also occur as a cost‐avoiding strategy by females (Westneat & Stewart, 2003). Harassment by extra‐pair males may be reduced in duration and intensity if a female complies with extra‐pair copulations that they might otherwise avoid participating in, which in other taxa like insects is known as convenience polyandry (Arnqvist & Rowe, 2005). Still, the rate of harassment relative to the rate of extra‐pair copulation is poorly described and among‐species investigations would be informative.

### (2) Mate guarding (M)

Mate‐guarding, where the male follows the female to avoid her engaging in extra‐pair copulations, occurs often in the wild (Birkhead & Møller, 1992; Harts, Booksmythe & Jennions, 2016). Yet, mate guarding potentially leads to physical conflict with other males (Birkhead, 1979), and risks physical harm, energetic costs (Low, 2006) and time (lost opportunity) costs (Komdeur, 2001) for the guarding male.

To study the energetic cost of mate‐guarding in Seychelles warblers *Acrocephalus sechellensis*, Komdeur (2001) experimentally manipulated the density of neighbouring males, hence changing the necessity of mate‐guarding. This study demonstrated a negative relationship between relative body mass (i.e. energetic state) and time spent mate‐guarding. Moreover, the experimentally induced reductions in mate guarding resulted in substantial increases in male relative body mass and time spent foraging, although this might also be due to reduced need for territory defence etc. Mate‐guarding was also suggested as being energetically costly for the stitchbird, where relative body mass is negatively affected by the number of extra‐pair male intruders (Low, 2006). Therefore, mate‐guarding can be a direct cost for males, as reductions in male body mass are expected to be associated with reduced survival (Komdeur, Bullock & Rands, 1991).

Although the extent of mate guarding varies (Birkhead & Møller, 1992; Low, 2006), ‘high‐quality’ males have been shown to perform less guarding in numerous species (Harts *et al*., 2016). This may be because females prioritize mating based upon the social male's relative ‘quality’ (Harts *et al*., 2016). Work on male attractiveness, which could be a proxy for male quality, also suggests that unattractive males guard more intensively than attractive males (Johnsen & Lifjeld, 1995; Dowling & Webster, 2017). Such a decrease in guarding with individual quality is also predicted by theoretical models (Kokko & Morrell, 2005). High quality males may have a higher ‘lost opportunity’ cost through mate guarding, because these high‐quality males could likely successfully seek extra‐pair copulations. Hence, high‐quality individuals are likely to exhibit both a lower absolute and relative cost of paternity assurance. However, measures of male ‘quality’ are often attributed to individual quality markers such as body size or sexually‐selected signals (Harts *et al*., 2016). This represents an among‐individual quality effect, but few studies have focused on the within‐individual quality effect of energetic state (i.e. relative body mass or fat reserves). It would be interesting to understand within‐ *versus* among‐individual cost‐variation and this warrants further investigations.

The costs of mate‐guarding can, however, be reduced by guarding only during the partner's fertile period (Krokene *et al*., 1996; Currie *et al*., 1998; Canal, Jovani & Potti, 2012). In several species, males guard their social partner throughout their fertile period and seek extra‐pair mating outside this period (Currie *et al*., 1998; Canal *et al*., 2012). Furthermore, the costs of mate‐guarding could be lowered by reducing the intensity of guarding behaviour. However, intensity of chasing potential conflicting males was independent of relative body mass in guarding male Seychelles warblers (Komdeur, 2001). This indicate that energetic state may affect only the extent of mate‐guarding, rather than the intensity of the behaviour itself (Komdeur, 2001). Interestingly, mate‐guarding could also have the by‐product benefit of increasing the partner's condition, because the presence of an alert partner could reduce the female's adaptive level of anti‐predator vigilance, which could enhance an indirect benefit of mate‐guarding (Abbey‐Lee *et al*., 2018). Although direct costs of mate‐guarding have been demonstrated, no studies have explored the potential indirect costs of mate‐guarding. If mate‐guarding reduces the energetic state of males in species with obligate biparental care, this might reduce potential for male care, with potential effects on offspring quality. If so, one might expect the extent of paternal care to covary with mate‐guarding, which can be manipulated both across individuals and across broods of the same individuals.

## V. CONCLUSIONS

(1) Studying the costs of extra‐pair behaviour is important for understanding the drivers of variation in extra‐pair behaviour, both in individuals and in species. Costs of extra‐pair behaviour likely plays a significant role in shaping extra‐pair behaviour. However, quantifying these costs is often difficult, and therefore difficult to compare. Future work should approach these questions interdependently of sex, and specific hypotheses should be tested empirically to support existing theory. Observational and descriptive studies would also be valuable, particularly on harassment (from both social and extra‐pair males) and STDs.
(2) As a result of extra‐pair paternity, males experience reduced paternity, while females can experience reduced parental care and reduced genetic quality as the most obvious costs. When it comes to extra‐pair copulations, there are search costs for both sexes, and from males trying to prevent loss of paternity. Costs associated with STDs are particularly poorly known.
(3) Female engagement in extra‐pair behaviour is still poorly understood despite many decades of research. We suggest that an improved understanding of costs (rather than empirical benefits) may improve our understanding, for example, in the context of sexual conflict hypotheses.
(4) In order to test these hypotheses precisely access to closed breeding populations with complete genetic pedigree data are likely required, and our work here further underpins the value of maintaining such long‐term study systems. Alternatively, captive breeding systems may be appropriate in some circumstances. Future work could seek to describe both the benefits and costs of specific extra‐pair behaviours (or related traits), partitioned by sex and mechanistic context.
